# A Novel Quantitative Parameter for Static Myocardial Computed Tomography: Myocardial Perfusion Ratio to the Aorta

**DOI:** 10.3390/jcm11071816

**Published:** 2022-03-25

**Authors:** Takanori Kouchi, Yuki Tanabe, Takumasa Takemoto, Kazuki Yoshida, Yuta Yamamoto, Shigehiro Miyazaki, Naoki Fukuyama, Hikaru Nishiyama, Shinji Inaba, Naoto Kawaguchi, Tomoyuki Kido, Osamu Yamaguchi, Teruhito Kido

**Affiliations:** 1 Department of Radiology, Graduate School of Medicine, Ehime University, Shitsukawa, Toon 791-0295, Japan; taka.xlay56@gmail.com (T.K.); take10toku6@gmail.com (T.T.); kn0wn951753@gmail.com (K.Y.); please_zantetsu@yahoo.co.jp (Y.Y.); n.fukuyama68@gmail.com (N.F.); nishiyama.hikaru.mj@ehime-u.ac.jp (H.N.); n.kawa1113@gmail.com (N.K.); tomozo0421@gmail.com (T.K.); terukido@m.ehime-u.ac.jp (T.K.); 2 Department of Cardiology, Pulmonology, Hypertension and Nephrology, Graduate School of Medicine, Ehime University, Shitsukawa, Toon 791-0295, Japan; shigehiro.miyazaki.0123@gmail.com (S.M.); inaba226@gmail.com (S.I.); yamaguti@m.ehime-u.ac.jp (O.Y.)

**Keywords:** computed tomography, computed tomography perfusion, myocardial perfusion abnormality

## Abstract

We evaluated the feasibility of myocardial perfusion ratio to the aorta (MPR) in static computed tomography perfusion (CTP) for detecting myocardial perfusion abnormalities assessed by single-photon emission computed tomography (SPECT). Twenty-five patients with suspected coronary artery disease who underwent dynamic CTP and SPECT were retrospectively evaluated. CTP images scanned at a sub-optimal phase for detecting myocardial perfusion abnormalities were selected from dynamic CTP images and used as static CTP images in the present study. The diagnostic accuracy of MPR derived from static CTP was compared to those of visual assessment and conventional quantitative parameters such as myocardial CT attenuation (HU) and transmural perfusion ratio (TPR). The area under the curve of MPR (0.84; 95% confidence interval [CI], 0.76–0.90) was significantly higher than those of myocardial CT attenuation (0.73; 95% CI, 0.65–0.79) and TPR (0.76; 95% CI, 0.67–0.83) (*p <* 0.05). Sensitivity and specificity were 67% (95% CI, 54–77%) and 90% (95% CI, 86–92%) for visual assessment, 51% (95% CI, 39–63%) and 86% (95% CI, 82–89%) for myocardial CT attenuation, 63% (95% CI, 51–74%) and 84% (95% CI, 80–88%) for TPR, and 78% (95% CI, 66–86%) and 84% (95% CI, 80–88%) for MPR, respectively. MPR showed higher diagnostic accuracy for detecting myocardial perfusion abnormality compared with myocardial CT attenuation and TPR.

## 1. Introduction

In coronary artery disease (CAD), it is important to assess the significance of myocardial ischemia to determine the optimal treatment strategy before revascularization [1,2]. In current practice, various myocardial perfusion imaging (MPI) techniques such as single-photon emission computed tomography (SPECT), magnetic resonance (MR) imaging, or positron emission tomography (PET) have been widely used [3,4,5,6]. Recently, developments in computed tomography (CT) technology have fulfilled the technical prerequisites for the application of stress myocardial CT perfusion (CTP) for the evaluation of CAD [7]. Two main techniques have been applied for myocardial CTP imaging: static CTP and dynamic CTP [8]. Static CTP is mainly evaluated by visual assessment, while dynamic CTP is assessed using several quantitative parameters derived from the time attenuation curve, which is advantageous for adapting to varying ischemic severities and for assessing the therapeutic effect of revascularization therapy with high objectivity and reproducibility [9,10]. However, dynamic CTP has a disadvantage; its radiation exposure is relatively high compared with that for static CTP (9.23 mSv vs. 5.93 mSv) [11]. Moreover, dynamic CTP requires wide detector coverage and high temporal resolution to obtain the data of the whole heart without temporal and spatial gaps [12]. Hence, a robust quantitative evaluation of static CTP imaging is required. Semi-quantitative parameters for static CTP imaging have been proposed, such as myocardial CT attenuation and transmural perfusion ratio (TPR), but the diagnostic accuracy of these parameters has been reported to be inferior to that of visual assessment for the detection of myocardial ischemia [13]. We introduced the myocardial perfusion ratio to the aorta (MPR) as a new quantitative parameter for static CTP and evaluated its feasibility for identifying myocardial perfusion abnormalities.

## 2. Materials and Methods

### 2.1. Study Population

This retrospective study was approved by our institution’s human research committee (registration number: 1810021). The need for informed consent was waived due to the retrospective nature of the study. Thirty patients from our cardiac database who underwent stress dynamic myocardial CTP and SPECT-MPI between February 2013 and March 2015 were enrolled in this study. In the present study, static myocardial CTP images were retrospectively extracted from dynamic myocardial CTP imaging data. The attending physician determined the indications for myocardial CTP and SPECT for the assessment of CAD due to effort angina via ST-T changes on electrocardiography, reduction in angina symptoms after administration of nitroglycerin, or multiple coronary risk factors. The exclusion criteria were as follows: (1) cardiomyopathy; (2) left ventricular ejection fraction <20%; (3) greater than first-degree atrioventricular block; (4) left complete bundle branch block; (5) valvular heart disease; (6) history of coronary artery bypass grafting; and (7) poor image quality of stress dynamic CTP and SPECT. The radiation exposure was calculated using the dose-length product with a conversion factor of 0.014, as described previously [14].

### 2.2. Dynamic Myocardial CTP Scan Protocol

An established dynamic myocardial CTP scan protocol was performed for this study [15]. All stress dynamic myocardial CTP scans were performed using a 256-slice multidetector row CT scanner (Brilliance iCT; Philips Healthcare, Cleveland, OH, USA) and an automatic dual injector (Stellant DualFlow; Nihon Medrad KK, Osaka, Japan). A timing bolus scan was performed to estimate the scan timing and contrast medium (CM) concentration for coronary CT angiography (CTA) using a 20% solution of the CM (iopamidol 370 mg iodine/mL; Bayer Yakuhin, Ltd., Osaka, Japan) diluted with saline (5.0 mL/s for 10 s), followed by a saline chaser (5.0 mL/s for 4 s) [16]. The timing bolus scan was performed with axial data acquisition at the level of the ascending aorta. Three minutes after stress loading via intravenous infusion of adenosine triphosphate (Adetphos-L KOWA injection 20 mg; Kowa Company Ltd., Tokyo, Japan; 0.16 mg/kg/min, for 5 min), a stress dynamic CTP scan was performed for 30 consecutive cardiac cycles with the prospective electrocardiography-gated dynamic mode, which targets a phase of 40% RR interval using a bolus of CM (50 mL, 5.0 mL/s for 10 s) followed by a saline chaser (5.0 mL/s for 4 s). The scan parameters for the timing bolus scan were as follows: tube current of 50 mA; tube voltage of 120 kVp; and collimation at 2 × 16 × 0.625 mm. The scan parameters of the dynamic CTP scan were as follows: tube current of 80 mAs; tube voltage of 100 kVp; and collimation at 64 × 1.25 mm. Subsequently, CTA was performed using a diluted CM followed by a saline chaser, as previously described [16].

### 2.3. Analysis of Aortic Peak Enhancement in Timing Bolus and Dynamic CTP Scans

The timing bolus scan and dynamic CTP scan data sets were transferred to a dedicated software (Synapse Vincent ver.5; Fujifilm Medical Systems, Tokyo, Japan). A radiological technologist (9 years of experience in cardiac imaging) independently set the regions of interest (ROI) within the ascending aorta and measured aortic peak enhancement (PE) for each timing bolus scan and dynamic CTP scan data.

### 2.4. Post-Processing and Image Analysis of Myocardial CTP Imaging

A series of dynamic CTP images were reconstructed using a 360° reconstruction algorithm. Elastic registration and a spatiotemporal filter were used to reduce the image noise spatially and temporally through a dedicated workstation (IntelliSpace Portal; Philips Healthcare, Amsterdam, The Netherlands). For all cases, one radiological technologist (9 years of experience in cardiac CT imaging) selected a sub-optimal phase from dynamic CTP images as static CTP images according to the results of a previous study [17]. A short-axis view from the base to the apex of the left ventricle with 5 mm thickness without overlap was obtained using multi-planar reformation.

**Figure 1:**
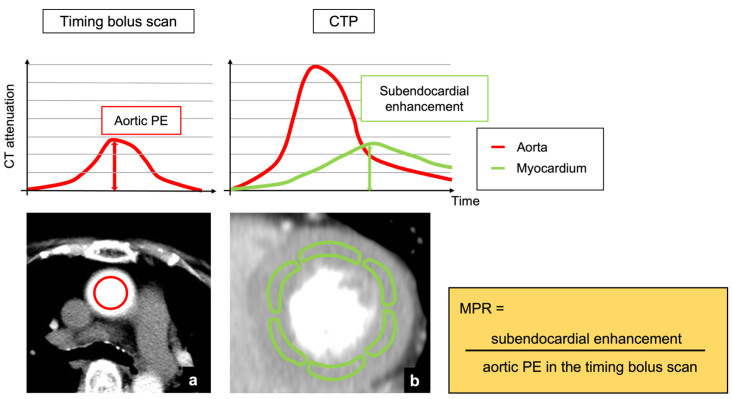
Analysis of MPR derived from myocardial CTP and timing bolus scan. (**a**) An axial image at the level of the ascending aorta in the timing bolus scan. (**b**) A short-axis view of left ventricle which was obtained from the static CTP image. PE was defined as the difference between baseline and peak CT attenuation of the aorta. A sub-optimal phase of dynamic CTP series was selected as static CTP image. MPR was calculated as follows: MPR = subendocardial enhancement/aortic PE in the timing bolus scan. CTP, computed tomography perfusion; PE, peak enhancement; MPR, myocardial perfusion ratio.

For qualitative assessment, one radiologist and cardiologist (6 years of experience in cardiac CT imaging each), both of whom were blinded to all other data, visually assessed all static CTP images to identify myocardial perfusion abnormalities as low-attenuation areas according to the 16-segment model [18]. The window width and level were arbitrarily adjusted to the optimal settings in each case. The final assessment was obtained through consensus. For quantitative assessment, another radiologist (7 years of experience in cardiac CT imaging) analyzed endocardial CT attenuation, TPR, and MPR using commercially available software (Synapse Vincent ver.5; Fujifilm Medical Systems, Tokyo, Japan) according to the 16-segment model [18]. The ROIs were set within both the endocardial and epicardial myocardium in each segment to calculate myocardial CT attenuation. MPR was defined as the endocardial CT enhancement of a specific segment divided by the PE of the ascending aorta in the timing bolus scan (Figure 1). TPR was defined as the endocardial CT attenuation of a specific segment divided by the mean of the epicardial CT attenuation of all segments [19]. To determine the inter-observer agreement of quantitative parameters, ten randomly selected patients were analyzed by a radiologist blinded to all other data (8 years of experience in cardiac CT imaging).

### 2.5. SPECT-MPI Scan Protocol and Image Analysis

A stress/rest SPECT-MPI was performed using a cadmium zinc telluride camera (Discovery NM 530c, GE Healthcare, Princeton, NJ, USA), as previously described [20]. Stress SPECT scans were performed 60 min after an injection of 99mTc-tetrofosmin (Myoview; Nihon Medi-Physics Co., Ltd., Tokyo, Japan) or 99mTc-sestamibi (Cardiolite; FUJIFILM RI Pharma Co., Ltd., Tokyo, Japan) at a dose range of 296–370 MBq. Four hours later, a rest SPECT scan was performed with 740 MBq of 99mTc myocardial perfusion agent. Cardiac long- and short-axis views were obtained using acquired perfusion data of the patients in the supine position.

Two radiologists (7 and 12 years of experience in SPECT-MPI), who were blinded to all other data, semi-quantitatively assessed stress and rest SPECT images using a 5-point scale (0 = normal, 1 = mildly reduced; 2 = moderately reduced; 3 = severely reduced, and 4 = absent). Discrepancies were resolved by consensus. A myocardial segment with a score ≥2 in the stress image was defined as an abnormal perfusion segment [21]. Reversible perfusion abnormality that was present in the stress state and resolved at rest state indicated ischemia. Fixed perfusion abnormality, which was present in both stress and rest states in the same segment, indicated infarction.

### 2.6. Statistical Analysis

Continuous variable data were expressed as mean (standard deviation) or median (25th–75th percentiles) based on the Shapiro–Wilk test results. The scan heart rates were compared during stress and resting CT using a paired *t*-test. The correlation in the aortic peak enhancement between the timing bolus and dynamic CTP scans was evaluated using Pearson’s correlation coefficient. The inter-observer agreements for visual assessment of static myocardial CTP and SPECT-MPI were assessed using the Cohen κ value. The inter-observer agreement for endocardial CT attenuation, TPR, and MPR was assessed using the interclass correlation coefficient (ICC). The endocardial CT attenuation, TPR, and MPR were compared between normal and abnormal perfusion segments using the Mann–Whitney U test. The diagnostic accuracy of visual assessment, endocardial CT attenuation, TPR, and MPR for detecting myocardial perfusion abnormality assessed by SPECT-MPI were analyzed by receiver operating characteristic curve analysis (ROC) and compared using Delong’s test [22]. The cut-off values of endocardial CT attenuation, TPR, and MPR for identifying myocardial perfusion abnormality were determined using Youden’s index. The sensitivity, specificity, positive predictive value, negative predictive value, and area under the receiver operating characteristic curve (AUC) with 95% confidence intervals were also analyzed for visual assessment, endocardial CT attenuation, TPR, and MPR. Statistical significance was set at *p* < 0.05. Statistical analyses were performed using JMP software (version 13.0; SAS Institute, Cary, NC, USA).

## 3. Results

### 3.1. Study Population

Of the 30 patients, 5 were excluded because of a history of coronary artery bypass grafting (*n* = 1), cardiomyopathy (*n* = 1), and poor image quality in dynamic CTP due to insufficient breath-hold (*n* = 3), with 25 patients finally enrolled. Table 1 shows patient characteristics. No patient experienced any cardiac events during their imaging session. The scan heart rate increased significantly from 65.4 (10.2) beats/min at rest to 80.0 (8.0) beats/min at stress CTP scans (*p* < 0.0001). The mean effective radiation doses for timing bolus scan and dynamic CTP were 75.1 (8.4) and 754.4 (0.7) (DLP), respectively. The total amount of CM used in the timing bolus scan and dynamic CTP was 59.1 (2.6) mL.

### 3.2. Characteristics of Myocardial Segments Assessed by SPECT-MPI

The interobserver agreement for perfusion abnormality on SPECT-MPI assessment was 0.83, and we concluded that the reliability was satisfactory (>0.70). Of the 400 segments, 63 were diagnosed as abnormal perfusion segments. Of the abnormal perfusion segments, 14 were diagnosed as ischemic segments and 49 were diagnosed as infarcted segments.

### 3.3. Aortic Peak Enhancement in Timing Bolus Scan and Dynamic CTP Scan

The aortic peak enhancements were 80.8 (18.1) HU in the timing bolus scan and 393.8 (91.7) HU in the dynamic CTP scan. There was a significant correlation between the aortic peak enhancement of the timing bolus and dynamic CTP scans (r = 0.84, *p* < 0.0001).

### 3.4. Comparisons in Endocardial CT Attenuation, TPR, and MPR between Normal and Abnormal Perfusion Segments

The interobserver agreement of the endocardial CT attenuation, TPR, and MPR were 0.96 (0.95–0.97), 0.84 (0.79–0.88), and 0.95 (0.94–0.97), respectively. The endocardial CT attenuations in normal and abnormal perfusion segments were 128 (112–144) and 106 (95–124) HU; TPR in normal and abnormal perfusion segments were 1.0 (0.9–1.0) and 0.9 (0.8–1.0); and MPR in normal and abnormal perfusion segments were 1.0 (0.9–1.1) and 0.7 (0.5–0.8). There were significant differences in endocardial CT attenuation, TPR, and MPR between the normal and abnormal perfusion segments (*p <* 0.0001).

### 3.5. Diagnostic Accuracy of Visual Assessment, Endocardial CT Attenuation, TPR, and MPR

**Figure 2:**
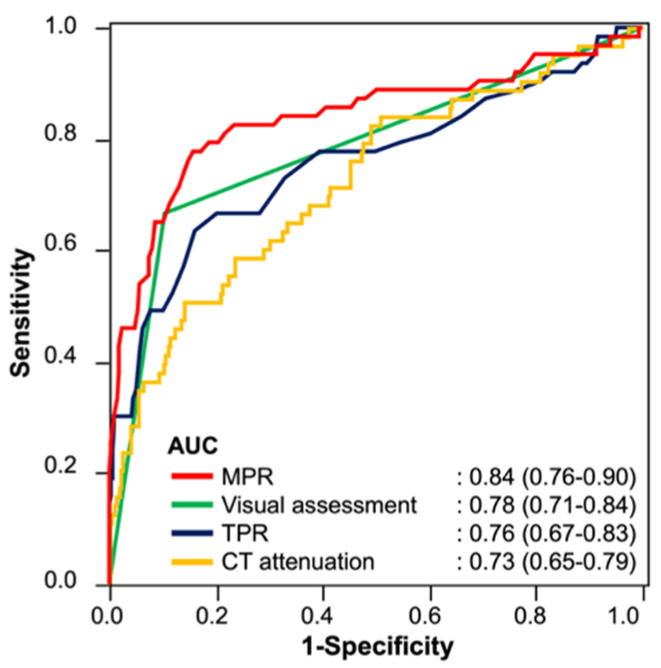
ROC curves of MPR, TPR, endocardial CT attenuation, and visual assessment for the detection of myocardial perfusion abnormality. The AUCs of MPR and visual assessment are comparable, and the AUC of MPR is significantly higher than those of TPR and endocardial CT attenuation. The 95% confidence intervals of the AUCs are shown in parentheses. ROC, receiver operating characteristic; MPR, myocardial perfusion ratio; TPR, transmural perfusion ratio; CT, computed tomography; AUC, area under the curve.

**Figure 3:**
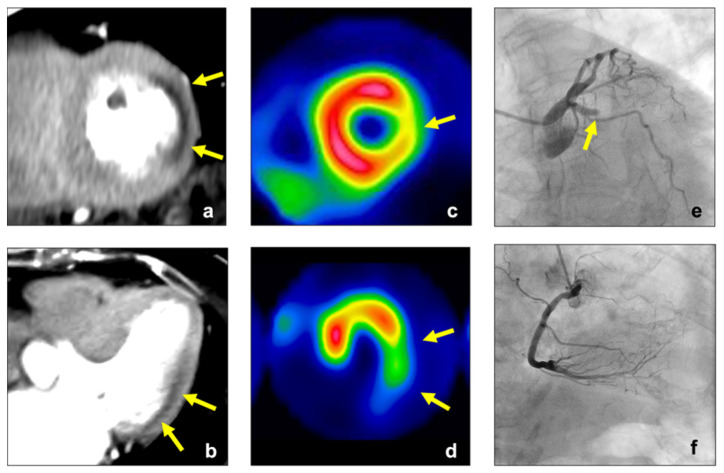
A 75-year-old woman with effort angina. Stress CTP image of the left ventricle showed a perfusion abnormality in the lateral wall (yellow arrow, (**a**,**b**)), and MPR in this lesion was lower than the cut-off value of MPR in this study (0.60 < cut-off value: 0.81). SPECT ((**c**,**d**), stress) showed a perfusion abnormality in the lateral wall (yellow arrow). ICA ((**e**), LCA, (**f**), RCA) revealed chronic total occlusion within the LCX. CTP, computed tomography perfusion; MPR, myocardial perfusion ratio; SPECT, single-photon emission computed tomography; ICA, invasive coronary angiography; LCA, left coronary artery; RCA, right coronary artery; LCX, left circumflex coronary artery.

**Figure 4:**
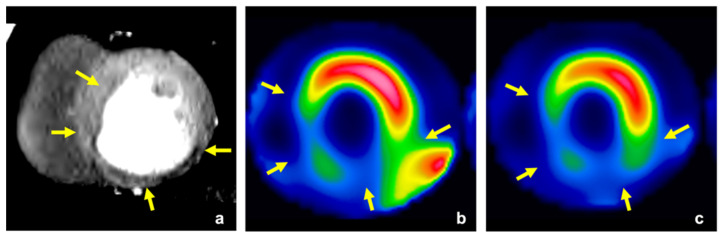
A 58-year-old man with effort angina. Stress CTP image of the left ventricle showed extensive myocardial perfusion abnormalities in the inferior, septal, and lateral inferior wall (yellow arrow, (**a**)). The perfusion abnormalities were detected with MPR (0.50 < cut-off value: 0.81), but not detected with TPR (0.96 > cut-off value: 0.92). SPECT ((**b**), stress; (**c**), rest) showed fixed perfusion abnormalities in these lesions, which indicated extensive old myocardial infarction (yellow arrow). CTP, computed tomography perfusion; MPR, myocardial perfusion ratio; TPR, transmural perfusion ratio; SPECT, single-photon emission computed tomography.

**Figure 5:**
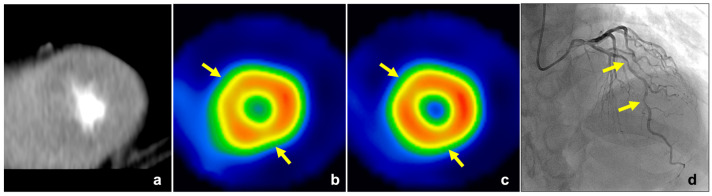
A 64-year-old man with effort angina. No obvious low-attenuation area was observed in the stress CTP image for the visual assessment (**a**), but the perfusion abnormalities were detected with MPR in the anterior and inferior myocardium of the apex (MPR: 0.69 and 0.71 < cut-off value: 0.81, respectively). SPECT ((**b**), stress; (**c**), rest) showed reversible perfusion abnormalities in the anterior and inferior myocardium in the apex (yellow arrow). ICA revealed tandem lesions with moderate and severe stenosis in the LAD (yellow arrow, (**d**)). The FFR of LAD was 0.68. CTP, computed tomography perfusion; MPR, myocardial perfusion ratio; SPECT, single-photon emission computed tomography; ICA, invasive coronary angiography; LAD, left anterior descending artery; FFR, fractional flow reserve.

The interobserver agreement for perfusion abnormality on visual CTP assessment was 0.74, and we concluded that the reliability was satisfactory (>0.70). The cut-off values of endocardial CT attenuation, TPR, and MPR were 106 HU, 0.92, and 0.81, respectively. The diagnostic accuracy for identifying myocardial perfusion abnormalities is summarized in Table 2. The sensitivity and specificity levels for detecting myocardial perfusion abnormalities were 67% (54–77%) and 90% (86–92%) for visual assessment, 51% (39–63%) and 86% (82–89%) for endocardial CT attenuation, 63% (51–74%) and 84% (80–88%) for TPR, and 78% (66–86%) and 84% (80–88%) for MPR, respectively. The AUC for identifying myocardial perfusion abnormality was 0.78 (0.71–0.84) for visual assessment, 0.73 (0.65–0.79) for endocardial CT attenuation, 0.76 (0.67–0.83) for TPR, and 0.84 (0.76–0.9) for MPR (Figure 2). The AUC of MPR was significantly higher than that of endocardial CT attenuation and TPR (*p* = 0.0013 for endocardial CT attenuation; *p* = 0.044 for TPR), while there was no significant difference in the AUC between MPR and visual assessment (*p* = 0.103). Representative clinical cases are shown in Figure 3, Figure 4 and Figure 5.

## 4. Discussion

The main findings of this study were as follows: (1) there were significant differences in MPR, myocardial CT attenuation, and TPR when comparing a myocardium with normal and abnormal perfusion; (2) the MPR had significantly higher diagnostic accuracy for detecting myocardial perfusion abnormality than myocardial CT attenuation and TPR; and (3) the MPR had higher sensitivity for detecting myocardial perfusion abnormality in comparison with visual assessment.

For static CTP imaging, myocardial CT attenuation is a simple quantitative parameter, but it is affected by various pathophysiological differences, such as the patient’s body weight and cardiac function [15]. Indeed, myocardial blood flow assessed by [^15^O] H_2_O PET was variable even in healthy people [23]. Tanabe et al. reported that these variations could be corrected by calculating the PE ratio of the myocardium to the aorta [24]. However, this correction method is not available for static CTP because it is impossible to accurately measure aortic PE. In this study, we corrected for myocardial CT enhancement with aortic PE obtained in a timing bolus scan with diluted CM, which was scanned for coronary CTA. Kawaguchi et al. reported that observed enhancement within coronary CTA was made uniform by adjusting the amount of CM based on the results of the timing bolus scan, despite individual differences regarding clinical background [15]. The aortic PE in dynamic CTP scan can be predicted using timing bolus scan data, and there was a good correlation in the aortic PE between the timing bolus and dynamic CTP scans in our results. Therefore, MPR could correct the variability of myocardial CT attenuation and showed higher diagnostic accuracy for detecting myocardial perfusion abnormalities than myocardial CT attenuation.

TPR is another quantitative parameter proposed in static CTP scans. TPR is a stable parameter that can be used to calculate the ratio of subendocardial CT attenuation to subepicardial CT attenuation [19]. Yang et al. reported that TPR had higher diagnostic accuracy for the detection of myocardial ischemia than myocardial CT attenuation, as replicated in the present results [13]. Furthermore, the MPR had a significantly higher diagnostic accuracy than TPR in our study. The reason for this was that TPR showed higher false-negative results compared with MPR. Ko et al. reported that TPR was falsely normalized in the presence of balanced transmural ischemia because both subendocardial and subepicardial CT attenuation were decreased [25]. In the present study, most of the false-negative results were observed in patients with extensive perfusion abnormalities. That being said, MPR is an enhancement ratio of the subendocardial myocardium to the ascending aorta; thus, it has a robust capability for detecting myocardial abnormalities even in cases with extensive perfusion abnormality.

Visual assessment is a standard method for the assessment of static myocardial CTP imaging. According to our results, the visual assessment had higher specificity and lower sensitivity than the MPR. Yuehua et al. also reported a low sensitivity and high specificity of visual assessment (62.7% and 97.7%, respectively) for detecting myocardial ischemia [26]. The reason for the high specificity in visual assessment is that it has the advantage of clarifying false perfusion abnormalities, such as beam hardening artifacts and motion artifacts. Visual assessment can identify these artifacts, which usually have a triangular shape, originate from the region of high attenuation next to it, and do not conform to vascular territories [27]. The reason for the low sensitivity in visual assessment is that it might miss mild perfusion abnormality due to the small difference in CT attenuation between normal and ischemic myocardium [26]. A previous animal study showed that the difference in myocardial CT attenuation between normal and ischemic myocardium was small in mild CAD [28]. Therefore, visual assessment requires an optimal adjustment of window width/level and substantial experience in myocardial CTP imaging. MPR allows for the quantitative assessment of myocardial perfusion using the cut-off value, which leads to lower dependence on the experience of observers than with visual assessment. Indeed, the MPR showed higher sensitivity for detecting myocardial perfusion abnormality than the visual assessment in the present study.

In the present study, we suggested the MPR as a novel quantitative parameter for static myocardial CTP imaging. MPR has the potential to reveal myocardial perfusion abnormalities that may be difficult for conventional quantitative parameters (e.g., myocardial CT attenuation, TPR) to detect. Then, MPR could be complementarily useful by utilizing the higher sensitivity for the cases in which myocardial perfusion abnormalities are not clearly detected by the visual assessment such as mild CAD in clinical practice. In a meta-analysis, myocardial CTP imaging had comparable diagnostic accuracy with conventional MPI (SPECT, MR, and PET) for detecting hemodynamically significant CAD [29]. Additionally, myocardial CTP imaging had some clinical advantages in comparison with these conventional MPI, such as higher accessibility, lower cost, and integration with coronary CTA [30,31]. Recently, new CT technologies such as ultra-high spatial resolution CT and photon-counting CT have been developed, which will lead to the further evolution of myocardial CTP imaging [32,33]. Myocardial CTP imaging has the potential to be widespread in future, and MPR will be one of the quantitative parameters for myocardial CTP imaging.

This study had several limitations. First, it was a retrospective, single-center study with a small sample size. Second, we did not evaluate coronary CTA because we focused on the feasibility of MPR for detecting myocardial perfusion abnormalities in static myocardial CTP imaging. Third, patients with myocardial infarction were not excluded from the present study population, which might have led to the lower diagnostic accuracy of TPR. However, myocardial CTP imaging will be performed in patients with myocardial infarction in the real world, especially in patients with unrecognized myocardial infarction. Finally, the static CTP images were derived from the dynamic CTP data in the present study, and the scan parameters might not be optimal for static CTP imaging. Further studies are required to evaluate the feasibility of MPR in the optimal setting for static CTP imaging.

In conclusion, MPR of the myocardium to the aorta using a timing bolus scan is a feasible quantitative parameter for assessing myocardial perfusion in static CTP imaging. MPR has a high diagnostic accuracy for detecting myocardial perfusion abnormalities, independent of substantial individual variations.

## Figures and Tables

**Table 1:** Patient characteristics.

Age (Years)	70.5 (9.5)
Men (%)	19 (76%)
Body mass index (kg/m^2^)	24.1 (3.1)
Coronary risk factors (number [%])	
Hypertension	18 (72%)
Dyslipidemia	12 (48%)
Diabetes mellitus	8 (32%)
Positive smoking history	16 (64%)
Family history of coronary artery disease	10 (40%)
HR (bpm)	
Baseline	65.4 (10.2)
Stress	80.0 (8.0)
Time periods between CT and SPECT (days)	27 (13–43)

Data are expressed as mean (standard deviation), median (interquartile range), or N (%). HR, heart rate; CT, computed tomography; SPECT, single-photon emission computed tomography.

**Table 2:** Diagnostic accuracy of MPR, TPR, endocardial CT attenuation, and visual assessment for detection of myocardial perfusion abnormality.

	Sensitivity (%)	Specificity (%)	PPV (%)	NPV (%)
MPR	78 (66–86)	84 (80–88)	48 (39–58)	95 (92–97)
TPR	63 (51–74)	84 (80–88)	43 (33–53)	92 (89–95)
CT attenuation	51 (39–63)	86 (82–89)	41 (30–52)	90 (87–93)
(HU)				
Visual	67 (54–77)	90 (86–92)	55 (43–65)	93 (90–96)
assessment				

Data are expressed as percentage (95% confidence interval). MPR, myocardial perfusion ratio; TPR, transmural perfusion ratio; CT, computed tomography; PPV, positive predictive value; NPV, negative predictive value.

## Data Availability

Data can be obtained from the corresponding author upon reasonable request.
